# Aggressive humor style and cyberbullying perpetration: Normative tolerance and moral disengagement perspective

**DOI:** 10.3389/fpsyg.2022.1095318

**Published:** 2022-12-23

**Authors:** Hong Zhu, Yilin Ou, Zimeng Zhu

**Affiliations:** School of Economics and Management, Tongji University, Shanghai, China

**Keywords:** aggressive humor style, cyberbullying perpetration, moral disengagement, online normative norms, tolerance for aggressive humor

## Abstract

The literature has acknowledged the correlation between aggressive humor style and cyberbullying perpetration; however, little is known about how this occurs. In this study, we sought to gain an understanding of how and when someone with an aggressive humor style may develop into a perpetrator of cyberbullying. We propose that whether an individual’s aggressive humor style results in cyberbullying perpetration depends on online social norms of tolerance for aggressive humor. When online normative tolerance for aggressive humor is high, individuals’ aggressive humor style is positively correlated with their moral disengagement, which, in turn, increases their intention to commit cyberbullying. When online normative tolerance for aggressive humor is low, the effect of individuals’ aggressive humor style on their moral disengagement is attenuated, which, in turn, weakens the relationship between aggressive humor style and cyberbullying perpetration. A total of 305 Chinese university students were recruited to participate in the experiment, and we found support for this hypothesis across the experiment. Several theoretical and practical implications are discussed.

## Introduction

Globally, cyberbullying is a major youth issue that involves intentionally inflicting harm or discomfort on another person through the use of the Internet, including social media (Lowry et al., 2016; Wang X. et al., 2019; Bai et al., 2020), which is becoming increasingly prevalent among adolescents. According to Vogels (2021), the majority of teenagers in 2021 who have experienced cyberbullying often suffer from depression and other mental problems. Therefore, to combat cyberbullying more effectively, it is vital to understand what motivates young people to bully others online (Varjas et al., 2010; Law et al., 2012; Steer et al., 2020). As noted in recent studies, aggressive humor might be a critical factor in the perpetration of cyberbullying (Wong and McBride, 2018; Steer et al., 2020; Maftei et al., 2022). In most studies, however, a relationship between aggressive humor and cyberbullying perpetration has been acknowledged or inferred in passing, but little empirical evidence has been provided to support this assertion. For example, Steer et al. (2020) found that humor-motivated cyber-banter or cyber-teasing may be associated with the perpetration of cyberbullying, with no further explanation, measurement, or modeling. Interestingly, Sari (2016) also suggested a link between an aggressive humor style and cyberbullying perpetration; however, it is less clear how and when perpetrators’ aggressive humor style has a significant effect on their cyberbullying perpetration. Thus, understanding the effect of aggressive humor on cyberbullying perpetration is especially pressing. Therefore, we sought to explore how and when perpetrators’ aggressive humor style might result in cyberbullying perpetration, as well as to extend previous investigations by exploring the mediating effect of perpetrators’ moral disengagement and the moderating effect of online normative tolerance for aggressive humor.

### Humor in online social interactions

A sense of humor often plays an important role in young people’s online social interactions (Burkley, 2022; Liao et al., 2022). Broadly defined, a sense of humor is a trait-like individual attribute characterized by behavior, attitude, or ability that facilitates amusement during social interactions (Martin, 2001). Humor is often used on social media as a means of promoting social cohesion (Yam et al., 2018). For example, Jones et al. (2021) argued that the use of humor in response to friends’ online postings is an effective method for maintaining positive relationships. However, not all forms of humor are positive. In fact, humoristic expressions can be used for a variety of social purposes—sometimes opposite ones. They may be used to strengthen relational bonds or to defuse awkwardness, but they may also serve to demonstrate superiority over others (Martin and Ford, 2018). Accordingly, Martin et al. (2003) attempted to better predict the behavior behind humor by dividing it into four subtypes: affiliative, self-enhancing, aggressive, and self-defeating. Affiliative humor promotes interpersonal bonds and reduces interpersonal tensions through benign and well-meant humor; in a manner similar to coping humor, self-enhancing humor involves maintaining a humorous outlook in the face of stress and adversity; self-defeating humor involves humiliating or making fun of oneself to gain the approval of others and avoid criticism from others; and aggressive humor is characterized by hostile, cynical, or sarcastic jokes, comments, teasing, or banter intended to denote superiority over others (boosting the self). Martin et al. (2003) also suggested that adaptive humor styles are often associated with positive outcomes [i.e., happiness (Ford et al., 2014) and social competence (Yip and Martin, 2006; Semrud-Clikeman and Glass, 2010)], while maladaptive humor styles are usually related to negative consequences [i.e., aggression (Baron and Ball, 1974; Ryan and Kanjorski, 1998) and deviance (Yam et al., 2018)].

### Aggressive humor and cyberbullying perpetration

Considering the close alignment between aggression humor and specific characteristics of cyberbullying perpetration, we decided to focus on the aggressive humor style of cyberbullying perpetrators. In previous studies, the relationship between aggressive humor and cyberbullying perpetration has been mentioned in passing, but no empirical evidence has been provided regarding how and when such a relationship exists. For example, Klein and Kuiper (2006) briefly described how people use aggressive humor on others to humiliate them, reduce their popularity, and gain superiority over them. Sari (2016) proved without theoretical modeling that adolescents use aggressive humor to provoke anger and humiliate their peers. Although it is widely accepted that there is a correlation between aggressive humor and cyberbullying perpetration, it is less clear how and when perpetrators’ aggressive humor style affects their cyberbullying behavior. Steer et al. (2020) called for future studies to pay closer attention to perpetrators’ moral disengagement mechanisms (i.e., something funny rather than harmful) when explaining the association between aggressive humor and cyberbullying, which is generally shaped by actions such as using technology to create funny photos of victims, creating websites with derogatory statements, or sending “funny” messages, e-mails, photos, or videos to victims and groups. Therefore, in response to Steer et al.’s (2020) call, we investigated how and when someone with an aggressive humor style would become a perpetrator of cyberbullying.

### Moral disengagement as a mediator

Moral disengagement theory describes psychological maneuvers as a means of selectively disengaging an individual’s self-regulation mechanisms so that adverse behaviors can be performed without psychological repercussions (Bandura et al., 1996; Bandura, 2002; Chan et al., 2022). Bandura (2002) argued that ethics and morality are important to individuals. The act of engaging in activities in accordance with moral standards brings satisfaction and a sense of self-worth, while the act of engaging in activities that are contrary results in psychological discomfort, cognitive dissonance, and self-shaming. Although this is true, morality regulation does not always provide a permanent internal control system that is subject to change due to factors outside its control, including individual and contextual factors. For example, a person who has an aggressive humor style might gain an appreciation for committing cyberbullying perpetration through observation of others’ acquiescence to, and even agreement with, their aggressive jokes and become aggressive online with their weaker peers as a result (Sari, 2016). In addition, individuals who have an aggressive humor style can change their beliefs regarding cyberbullying perpetration and develop high moral disengagement that allows them to justify, rationalize, or neutralize their online aggression (Steer et al., 2020). In other words, individuals’ humor style may affect their level of moral disengagement and, in turn, influence their intention to commit cyberbullying. In line with this theoretical lens, a growing number of studies have established that moral disengagement mediates the association between individual factors (e.g., emotion-related personality and humor style) and cyberbullying perpetration (Ciucci and Baroncelli, 2014; Maftei and Măirean, 2023). To our knowledge, no study has examined the mediating role of moral disengagement in the effect of aggressive humor and cyberbullying perpetration, although, based on previous work, we expect there to be one. In the following section, we discuss two reasons why moral disengagement is an appropriate mediator.

First, individuals who use aggressive humor are more likely to develop an extreme sense of moral disengagement. According to moral disengagement theory, moral disengagement is a result of the growing interaction between their internal factors, such as experience and habit, and their external factors, such as social context (Bandura et al., 1996; Bandura, 2002). In other words, individuals’ moral disengagement can be shaped by their previous experience and language habits as a malleable cognitive orientation (Zhao et al., 2019). Many empirical studies support this argument, showing that young people’s moral disengagement is influenced by various factors, including their own language habits, previous experiences, emotions-related personality traits, and humor styles (Ciucci and Baroncelli, 2014; Paciello et al., 2021; Maftei et al., 2022; Maftei and Măirean, 2023). For example, individuals with a high level of aggressive humor in their language communication habits might increase their likelihood of activating a moral disengagement process for online sexist memes (Paciello et al., 2021). Thus, we assume that individuals with an aggressive humor style score higher on the moral disengagement scale.

Second, many cross-sectional studies have confirmed that those with a high level of moral disengagement are more likely to engage in cyberbullying, demonstrating that moral disengagement is positively related to cyberbullying, even after adjusting for third variables (Bussey et al., 2015; Allison and Bussey, 2017; Meter and Bauman, 2018; Orue and Calvete, 2019). There is also evidence from three longitudinal studies indicating that moral disengagement plays a significant role in predicting individuals’ cyberbullying perpetration (Marin-Lopez et al., 2020; Zhang et al., 2021; Yang et al., 2022). For example, Yang et al. (2022) indicated that there is a longitudinal relationship between moral disengagement and cyberbullying perpetration regarding peer pressure. It is important to note the positive associations between moral disengagement and cyberbullying perpetration in meta-analyses (Gini et al., 2014; Kowalski et al., 2014; Chen et al., 2017). In light of previous studies, we propose the following hypotheses:

*H1a*: Individuals’ aggressive humor style is positively related to their moral disengagement.

*H1b*: Moral disengagement is positively related to cyberbullying perpetration.

*H1c*: Individuals’ moral disengagement mediates the relationship between aggressive humor and cyberbullying perpetration.

### Moderation effects of normative tolerance for aggressive humor

It is possible to observe a style of humor that is aggressive along with social norms that tolerate the use of aggressive humor (Ford et al., 2001). Social norms of tolerance for aggressive humor may have a significant impact on how aggressive humor affects their justification and intention to engage in cyberbullying. An independent study demonstrated that the social norm of tolerating sexist humor is significantly associated with aggression in external behavior (Ford et al., 2001). That study focused on a specific type of aggressive humor, namely, sexist humor, which limits its validity as a generalization. Although previous research has shown that normative tolerance for aggressive humor predicts individuals’ behavior related to aggression activities (Ford et al., 2001; Allison et al., 2019; Paciello et al., 2021; Maftei and Măirean, 2023), to date, no study has examined whether normative tolerance for aggressive humor is significantly related to cyberbullying perpetration and rationalization and justification of aggressive and abusive behavior. In addition, it remains unclear whether normative tolerance for aggressive humor can significantly exacerbate the detrimental effects of aggressive humor on moral disengagement and cyberbullying.

According to moral disengagement theory, individuals’ moral cognition and behavior are a function of the interaction of individual and context factors (Bandura et al., 1996; Bandura, 2002). Personal and social influences play a joint role in shaping individuals’ moral judgments and actions (Wang X. et al., 2019). It has been suggested, for example, that aggression associated with sexism results from exposure to sexist humor and social norms, such as the normative tolerance for sexist humor (Ford et al., 2001). Therefore, we hypothesized that the interaction between individuals with an aggressive humor style and online normative tolerance for such humor would significantly affect their moral disengagement and involvement in cyberbullying. In particular, a high degree of tolerance for aggressive humor online is associated with an increase in individuals with aggressive humor style changing their beliefs regarding cyberbullying perpetration, evaluating aggressive behavior as morally acceptable, and making them develop moral disengagement that permits them to justify their abusive behavior. Therefore, if online social norms of tolerance for aggressive humor are high, individuals with aggressive humor may be more likely to perpetrate cyberbullying. One study roughly supported our assumption by indicating that sexist humor may impact male participants’ self-directed negative affect and behavior in response to sexual abuse (Paciello et al., 2021). Building on previous work, we propose the following hypothesis:

*H2*: The indirect effect of individual’s aggressive humor style on cyberbullying perpetration, via moral disengagement, is moderated by online normative tolerance for aggressive humor such that the indirect effect is stronger when online normative tolerance is high, but weakens when online normative tolerance is low.

### The present study

**Figure 1:**
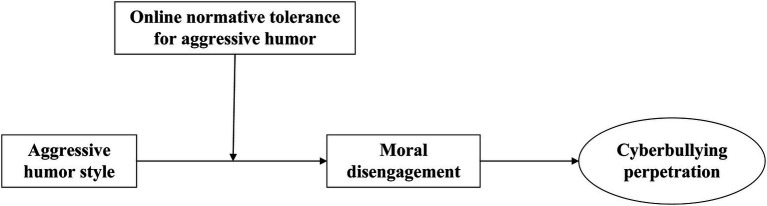
The proposed research model.

Our objective was to propose a more comprehensive understanding of the mechanism underlying the relationship between aggressive humor and cyberbullying perpetration according to moral disengagement theory. Specifically, we investigated moral disengagement as a mediator and online normative tolerance for aggressive humor as a moderator of such a relationship. As shown in Figure 1, we proposed a moderated mediation model to answer two questions: How does an aggressive humor style lead individuals to commit cyberbullying perpetration? Why?

## Materials and methods

### Participants

Three hundred and five participants were recruited from different universities in China, of which 55.74% were male and 44.26% were female. The age of the participants ranged from 16 to 25. Their average age was 20.81, with an average of 2.58 h per day spent on social media. Approximately 20 min were spent by each subject completing the entire experiment.

### Procedure and experimental design

We employed a factorial design and manipulated aggressive humor style (presence or absence). At the beginning of this study, participants were informed that the purpose of our study was to investigate the link between aggressive humor style and cyberbullying perpetration. Then, the participants were provided with a scenario followed by aggressive humor manipulation. The scenario was adapted from Kuiper et al. (2010). Participants were randomly assigned to one of two experimental conditions. In both conditions, participants were asked to read the following statements:

Imagine that you are having a snack in the cafeteria of your university with a friend from your class. It is about once every week that you meet this friend outside of class and spend some time discussing various personal matters. You announce to your casual friend today that the person you have been dating for the past year is seriously considering ending the relationship.

To manipulate aggressive humor style, we provide an example of aggressive humor for participants and also instructed them to imagine such scenario involving aggressive humor. Participants in the aggressive humor condition were asked to read the following statements:

When you share your dating relationship problems with your casual friend, he or she responds by making sarcastic and critical remarks about you. It is normal for a casual friend to make humorous comments that ridicule your performance and abilities. Using this type of putdown humor shows that your casual friend often expresses humor without considering its potential impact on other individuals.

To manipulate the control condition, we asked participants to read the following statements:

Your casual friend responds with humorous comments when you describe your dating relationship problems, but his or her humor is not sarcastic and critical. Using such gentle humor conveys the idea that your casual friend often expresses humor while considering its potential impact on others.

Following previous studies (Mayer et al., 2012; Barlow et al., 2018; Qin et al., 2020), participants completed a filler task that appeared unrelated (describing their hobby or favorite color). The manipulation check is then conducted using the aggressive humor scale (Martin et al., 2003; Kuiper et al., 2010; Yam et al., 2018; Evans et al., 2019). We matched each scenario with a manipulation question to avoid priming the subjects (Choi et al., 2018). Data from those who failed the manipulation check were excluded from the analysis if the manipulation check failed (Oppenheimer et al., 2009). After that, participants completed measures of cyberbullying perpetration intention, moral disengagement, and reported demographic information. The final data set consisted of 305 participants who answered one scenario each. Among these participants, 152 participants participated in the aggressive humor condition, whereas 153 participants participated in the control condition.

### Measures

#### Normative tolerance for aggressive humor

We measured normative tolerance for aggressive humor on a seven-point scale adapted from Ford et al.’s (2001). We made several adjustments to reflect the state nature of this construct, and asked participants to rate the extent to which they thought others would tolerate such humor of this type in the scenario described. A sample item is “Given the scenario just described, please indicate how critical others would be of those remarks (the main character) were highly correlated.” The Cronbach’s alpha coefficient for this construct in our sample was 0.839.

#### Moral disengagement

We measured moral disengagement using 16-item scale adapted from Bandura et al. (1996). This scale has been widely used to capture individuals’ moral disengagement in cyberbullying episodes (e.g., Chan et al., 2022; Yang et al., 2022). A sample item is “Online comments with aggressive jokes is just a way of joking.” The participants rated all items on a seven-point scale. The Cronbach’s alpha coefficient for this construct in our sample was 0.951.

#### Cyberbullying perpetration

We measured cyberbullying perpetration using nine-item Cyberbullying Scale adapted from Wright et al. (2015). Several studies have demonstrated the validity and reliability of this scale in the context of Chinese adolescents (Wang et al., 2016; Wang G.-F. et al., 2019). A sample item is “how often would the main character in the scenario say nasty things to someone or called them names using texts or online messages.” A seven-point scale was used to rate all items. The Cronbach’s alpha coefficient for this construct in our sample was 0.908.

#### Manipulation check for aggressive humor

The participants were asked to rate the aggressive humor style of the main character using a nine-item scale developed by Martin et al. (2003), Kuiper et al. (2010), Yam et al. (2018), and Evans et al. (2019). A seven-point scale was used to rate all items. Participants in the aggressive humor condition reported a significantly higher score (M = 4.29) than those in the control condition [M = 2.28, *t*(303) = −24.16, *p* < 0.001]. As a result, aggressive humor is manipulated.

### Data analysis

Four steps were involved in the data analysis. First, we carried out the analysis of correlations to examine Hypothesis1a,1b. Second, we examined the mediation effect proposed in Hypothesis 1c by SPSS PROCESS macro [model 4; Hayes, 2012]. Third, to examine the moderation effect proposed by Hypothesis 2, the PROCESS marco (Model 7) developed by Hayes (2012) was adopted. Finally, the bootstrapping method (Hayes, 2012) was also employed for the analysis of indirect effects.

## Results

### Descriptive analysis

Table 1 summarizes descriptive statistics and correlations. There is a significantly positive correlation between aggressive humor style and moral disengagement (r = 0.69, *p* < 0.01). In addition, there was a significant upward correlation between moral disengagement and cyberbullying perpetration (r = 0.74, *p* < 0.01). Besides, aggressive humor style was positively correlated with cyberbullying perpetration (r = 0.59, *p* < 0.01). Additionally, online normative tolerance was positively correlated with both moral disengagement (r = 0.45, p < 0.01) and cyberbullying perpetration (r = 0.41, *p* < 0.01).

**Table 1:** Descriptive statistics and correlations.

	M	SD	1	2	3	4	5	6
Gender	0.44	0.50	1					
Age	20.81	0.92	−0.12^*^	1				
Aggressive humor style	3.77	1.07	0.02	0.04	1			
Cyberbullying perpetration	3.05	1.11	−0.06	0.13^*^	0.59^**^	1		
Moral disengagement	3.40	1.21	−0.09	0.13^*^	0.69^**^	0.74^**^	1	
Online normative tolerance	3.43	1.23	0.03	−0.00	0.57^**^	0.41^**^	0.45^**^	1

*N* = 305. For gender, 1 = male, 0 = female.

* *p* < 0.05;

** *p* < 0.01.

### Testing for mediation effect

**Figure 2:**
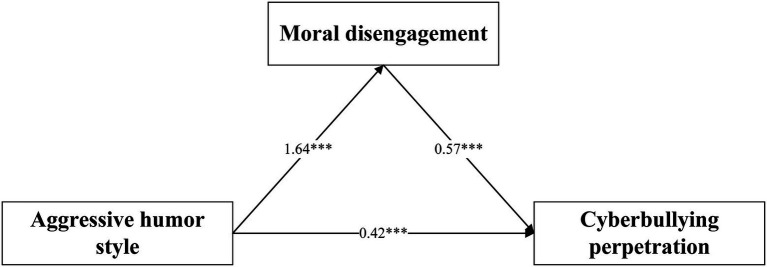
Testing for mediation effect. ^***^Significant at the 0.001 level; Unstandardized betas are reported.

As presented in Table 2, there was a significant correlation between aggressive humor style and moral disengagement (β = 1.64, *p* < 0.001, see Model 1 of Table 2). A significant correlation of moral disengagement and cyberbullying perpetration was also present (β = 0.57, *p* < 0.001, see Model 2 of Table 2). Thus, Hypothesis 1a and 1b were supported. Besides, Bootstrapping results showed an indirect effect, such that aggressive humor style increased cyberbullying perpetration intentions through moral disengagement (b = 0.93, 95% CI [0.69, 1.17]). Thus, moral disengagement mediated the effect of aggressive humor style on cyberbullying perpetration (see Figure 2), supporting Hypothesis 1c.

**Table 2:** Regression results.

Predictors	Model 1		Model 2	
	Moral disengagement		Cyberbullying perpetration	
	Β	t	β	t
Gender	0.01	0.10	−0.06	−0.73
Age	−0.04	−0.66	−0.06	−1.25
Aggressive humor style	1.64^***^	16.06	0.42^***^	3.62
Moral disengagement			0.57^***^	11.82
R^2^	0.46^***^		0.57^***^	
F	86.87		98.42	

*N* = 305.

* *p* < 0.05;

** *p* < 0.01;

*** *p* < 0.001.

### Testing for moderated mediation effect

**Figure 3:**
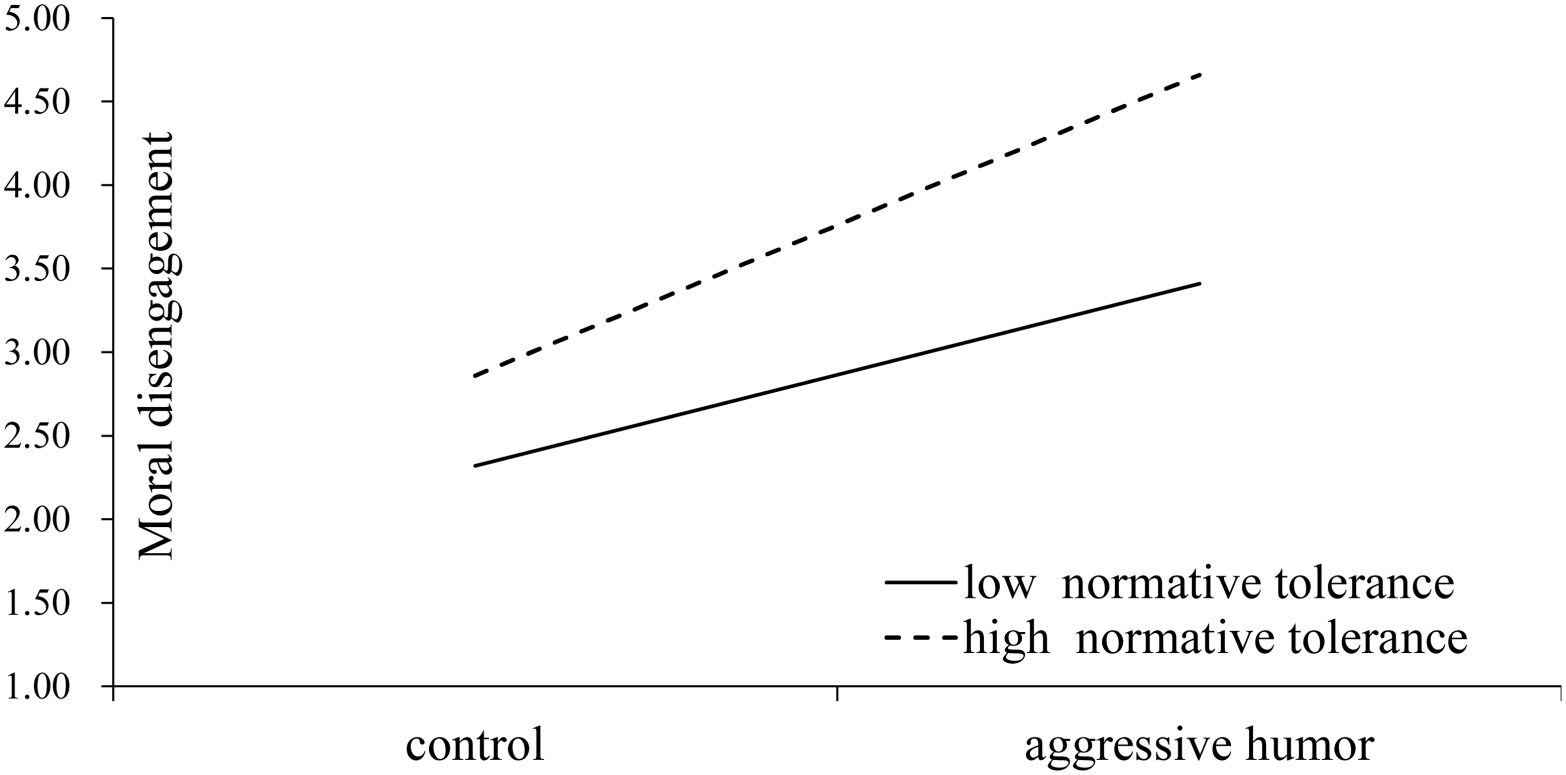
Joint effect of aggressive humor and online normative tolerance on moral disengagement.

In Table 3, the interaction between aggressive humor style and online normative tolerance significantly predicted moral disengagement [β = 0.29, *p* < 0.001, see Model 1 of Table 3, R^2^ = 0.60, *F*(1, 301) = 90.24]. It was also found that the indirect effect of aggressive humor on cyberbullying perpetration *via* moral disengagement varied significantly according to the moderator (online normative tolerance for aggressive humor), with an index of moderated mediation of 0.16, 95% CI [0.0659, 0.2631]. When online normative tolerance was low (high), there was a significant indirect effect of individuals’ aggressive humor style on their cyberbullying intention, such that aggressive humor style increased the likelihood of intentions to commit cyberbullying through increased moral disengagement (b = 0.61; 95% CI [0.4101, 0.8484], b = 1.02; 95% CI [0.7558, 1.2989], respectively). In order to better understand the results, we employed a figure which illustrates the combined effects of aggressive humor and online normative tolerance on moral disengagement (Figure 3). Our study examined indirect effects at high and low levels of the moderator (1 SD above and below) by constructing confidence intervals (Edwards and Lambert, 2007; Qin et al., 2020). Figure 3 showed a stronger positive relationship between aggressive humor style and moral disengagement when online normative tolerance was high (b_high_ = 1.80, t = 15.25, *p* < 0.001) compared to when it was low (b_low_ = 1.08, t = 7.79, *p* < 0.001). Thus, we concluded that the indirect effect of individuals’ aggressive humor style on cyberbullying perpetration, *via* moral disengagement, is moderated by online normative tolerance for aggressive humor such that the indirect effect is stronger when online normative tolerance is high, but weakens when online normative tolerance is low, supporting Hypothesis 2.

**Table 3:** Regression results.

Predictors	Model 1		Model 2	
	Moral disengagement		Cyberbullying perpetration	
	β	t	β	t
Gender	−0.01	−0.14	−0.06	−0.73
Age	−0.01	−0.24	−0.06	−1.24
Aggressive humor style	0.51	1.90	0.42^***^	3.61
Online normative tolerance	0.22^***^	4.45		
Aggressive humor style * Online normative tolerance	0.29^***^	3.92		
Moral disengagement			0.57^***^	11.82
R^2^	0.60^***^		0.57^***^	
F	90.24		98.42	

*N* = 305.

* *p* < 0.05;

** *p* < 0.01;

*** *p* < 0.001.

## Discussion

We proposed a moderated mediation model to explore how and when individuals with an aggressive humor style could become perpetrators of cyberbullying. In particular, we investigated whether online normative tolerance for aggressive humor moderates an indirect connection between aggressive humor style and cyberbullying perpetration *via* moral disengagement. We found compelling evidence that the impact of aggressive humor style on cyberbullying perpetration can be explained in part by moral disengagement. This indirect relationship was further moderated by online normative tolerance for aggressive humor. When there is high online normative tolerance for aggressive humor, those with an aggressive humor style are more likely to adopt a moral disengagement approach to perpetrate cyberbullying, while this effect may be attenuated in the case of low online normative tolerance for aggressive humor. This phenomenon can be explained in several different ways. First, based on moral disengagement theory, social norms of tolerance are believed to contribute to individuals rationalizing or justifying their adverse behavior without experiencing psychological repercussions (Kong and Yuan, 2018; Paciello et al., 2020). No matter what type of aggressive humor they display, individuals with a high perception of online normative social norms of tolerance for aggressive humor exhibit a much stronger belief that cyberbullying should be permitted than those with a low perception of online normative tolerance for aggressive humor. It is possible that because individuals who perceive a low level of normative tolerance for aggressive humor online are more likely to realize that it is inappropriate to engage in cyberbullying, the impact of an aggressive humor style may be limited (Pabian et al., 2016; Maftei and Măirean, 2023) A high level of online normative tolerance for aggressive humor, however, is more likely to result in the belief that moderate deviance or aggression is automatically permitted (Piccoli et al., 2020; Wachs et al., 2021). Second, individuals who perceive a high level of normative tolerance for aggressive humor are more likely to believe that aggressive behavior is acceptable. In contrast, individuals who perceive low levels of normative tolerance for aggressive humor are more likely to believe that aggressive behavior is not permitted, forming a striking contrast between these two groups (Harper, 2019; Mishna et al., 2020). As a result, individuals who perceive a high level of normative tolerance for aggressive humor may be more susceptible to the influence of aggressive humor styles.

### Theoretical contributions

This study makes several theoretical contributions to the extant literature on cyberbullying. First, this work contributes to cyberbullying literature by providing a richer understanding through the lens of moral disengagement of how and when an individual with an aggressive humor style would develop into a perpetrator of cyberbullying. Previous literature has mainly focused on examining the correlation between the aggressive humor style and cyberbullying perpetration (Sari, 2016; Qodir et al., 2019; Maftei and Măirean, 2023), and surprisingly few have explored whether individuals’ moral disengagement mechanisms are responsible for this indirect effect. Taking the moral disengagement viewpoint into consideration, it remains to be determined how and when someone with an aggressive humor style can influence their moral disengagement mechanism and ultimately, trigger their intention to commit cyberbullying. In light of moral disengagement theory (Bandura et al., 1996; Lo Cricchio et al., 2021; Chan et al., 2022), we provide one of the first, if not the first, insights into the causal mechanism underlying the relationship between aggressive humor style and cyberbullying perpetration, which contributes to understanding how and when an aggressive humor style would have an impact on adolescents’ cyberbullying perpetration.

Second, this paper contributes to cyberbullying literature by revealing a boundary condition on the effect of aggressive style on cyberbullying perpetration. In particular, we explored online normative tolerance for aggressive humor as the key boundary condition to achieve a more comprehensive understanding of the impact of aggressive humor on cyberbullying perpetration. As the literature on the relationship between aggressive humor and cyberbullying perpetration is still in its infancy (Dynel, 2021; Maftei and Măirean, 2023), it is vital to understand when the aggressive humor style has a significantly positive effect on cyberbullying and when such an effect is attenuated, so that we gain a better understanding of the boundary conditions that determine the impact of aggressive humor style. In this work, we revealed that an aggressive humor style contributes significantly to cyberbullying perpetration when individuals perceive online normative tolerance for such humor to be high. However, such positive influences would be diminished if individuals perceived online normative tolerance for such humor to be low. Therefore, these findings provide us with a deeper understanding of how aggressive humor impacts cyberbullying, showing that it is not static, but could be attenuated if online normative tolerance for such humor is low.

### Practical contributions

This work makes several practical contributions. First, the findings underscore the crucial role of aggressive humor in cyberbullying, prompting platform owners to pay more attention to users’ online interactions involving this type of humor. To prevent cyberbullying perpetration and improve the overall online climate, platform owners could employ machine learning techniques to detect users’ online posts involving aggressive humor, classify the posts into benign or potentially malicious categories, and set up automatic alerts in the latter category. Furthermore, platform owners could organize educational and training programs on topics that include what is appropriate humor for online interaction and what kinds of humor may hurt others, which helps distinguish between good and bad humor and mitigates the potential negative consequences of aggressive humor.

Second, aggressive humor is associated with moral disengagement, whereas moral disengagement contributes to cyberbullying. The concept of moral disengagement implies that cyberbullying perpetrators escape moral evaluation without experiencing cognitive dissonance by justifying their online posts as mere jokes (Barlett et al., 2021; Falla et al., 2021; Maftei et al., 2022). Teachers and parents should pay closer attention to children who have aggressive humor styles to prevent the emergence of this moral disengagement mechanism. They could educate these children on how to use polite jokes and provide examples showing how aggressive jokes may result in harm to others. It may also be beneficial for teachers to employ cyberbullying intervention programs that consider the elements of aggressive humor and moral disengagement when conducting cyberbullying intervention strategies.

Third, it has been suggested that a high level of online normative tolerance for aggressive humor may strengthen the impact of aggressive humor on cyberbullying perpetration, which is a reminder that platform owners should take steps to guide online social norms. There are some ways to guide online social norms of tolerance for aggressive humor (Abrams and Scheutz, 2022). For example, it would be helpful if platform owners played a film to educate users to use humor wisely and promote a positive ethos in the online community (Nabila et al., 2021; Rinaldi, 2021). Similarly, campaigns or educational lectures can be designed to guide online social norms of tolerance for aggressive humor so that individuals with aggressive humor will not be able to rationalize their cyberbullying behavior (Polanin et al., 2021; Lan et al., 2022).

### Limitations and future research

This study is one of the first—if not the first—to empirically examine how aggressive humor contributes to cyberbullying. Although the study provides a starting point, there are several limitations that future research should address. First, this work was designed to test a sample that is representative of the general situation of Chinese Middle school individuals. However, the sample might not perfectly represent adolescents worldwide. Hopefully, the findings of this study will be demonstrated more adequately in the future with samples from all around the world.

Second, we encourage future researchers to explore the impact of different humor styles, such as self-enhancing humor or self-defeating humor, on cyberbullying perpetration. We examined only the role of aggressive humor in cyberbullying perpetration because cyberbullying is perpetrated in a socially maladaptive (i.e., aggressive) style as opposed to a socially adaptive (i.e., affiliative) style, and the majority of cyberbullying events occur in an aggressive manner compatible with an aggressive humor style (Cuadrado-Gordillo and Fernández-Antelo, 2019; Steer et al., 2020). However, it is important to examine the role of other humor styles in cyberbullying perpetration, as an individual may have more than one style of humor (Schermer et al., 2017; Heintz and Ruch, 2019).

Finally, we recommend that future cyberbullying researchers take a temporal perspective into account when examining the role of aggressive humor style in cyberbullying perpetration. It is possible that some people with aggressive humor become aware of the negative outcomes of their humor style and change accordingly over time (Tsai et al., 2021). One likely outcome is that bystanders might find words involving aggressive humor offensive and support the target, so individuals with aggressive humor might vary their expressions of humor toward different people based on online normative tolerance for such humor (Mulvey et al., 2016; Thomas et al., 2020; Katz et al., 2022). Due to the complexity of these effects, we recommend that future researchers employ longitudinal studies as the best way to capture these effects.

## Conclusion

Overall, this study contributes to the cyberbullying literature by investigating how and when aggressive humor may lead to cyberbullying. Moreover, a mediating mechanism with moral disengagement as a key component was described. Furthermore, this study identified a boundary condition by showing how online normative tolerance for aggressive humor moderates the relationship between aggressive humor and moral disengagement, and an indirect relationship between aggressive humor and cyberbullying perpetration *via* moral disengagement. To conclude, the theoretical model developed in this paper provides empirical support for further research into how individuals with aggressive humor can perpetrate cyberbullying.

## Data availability statement

The raw data supporting the conclusions of this article will be made available by the authors, without undue reservation.

## Ethics statement

The studies involving human participants were reviewed and approved by Tongji University. The Ethics Committee waived the requirement of written informed consent for participation.

## Author contributions

HZ and YO designed the research. HZ collected the data and wrote the manuscript. YO and ZZ proofread the manuscript. All authors contributed to the article and approved the submitted version.

## Conflict of interest

The authors declare that the research was conducted in the absence of any commercial or financial relationships that could be construed as a potential conflict of interest.

## Publisher’s note

All claims expressed in this article are solely those of the authors and do not necessarily represent those of their affiliated organizations, or those of the publisher, the editors and the reviewers. Any product that may be evaluated in this article, or claim that may be made by its manufacturer, is not guaranteed or endorsed by the publisher.
